# Identification of genetic polymorphisms modulating nausea and vomiting in two series of opioid-treated cancer patients

**DOI:** 10.1038/s41598-019-57358-y

**Published:** 2020-01-17

**Authors:** Francesca Colombo, Giulia Pintarelli, Antonella Galvan, Sara Noci, Oscar Corli, Frank Skorpen, Pål Klepstad, Stein Kaasa, Alessandra Pigni, Cinzia Brunelli, Anna Roberto, Rocco Piazza, Alessandra Pirola, Carlo Gambacorti-Passerini, Augusto Tommaso Caraceni

**Affiliations:** 1 Fondazione IRCCS Istituto Nazionale Tumori, Milan, Italy; 2 Pain and Palliative Care Research Unit, IRCCS Istituto di Ricerche Farmacologiche Mario Negri, Milan, Italy; 3 European Palliative Care Research Center, Norwegian University of Science and Technology, Trondheim, Norway; 4 Department of Clinical and Molecular Medicine, Faculty of Medicine and Health Sciences, Norwegian University of Science and Technology, Trondheim, Norway; 5 Department of Anesthesiology and Intensive Care Medicine, St. Olavs University Hospital, Trondheim, Norway; 6 Department of Oncology, St. Olavs University Hospital, Trondheim, Norway; 7 Department of Medicine and Surgery, University of Milano-Bicocca, Monza, Italy

**Keywords:** Genotype, Next-generation sequencing

## Abstract

Nausea and vomiting are often associated with opioid analgesia in cancer patients; however, only a subset of patients develop such side effects. Here, we tested the hypothesis that the occurrence of nausea and vomiting is modulated by the genetic background of the patients. Whole exome sequencing of DNA pools from patients with either low (n = 937) or high (n = 557) nausea and vomiting intensity, recruited in the European Pharmacogenetic Opioid Study, revealed a preliminary association of 53 polymorphisms. PCR-based genotyping of 45 of these polymorphisms in the individual patients of the same series confirmed the association for six SNPs in *AIM1L, CLCC1, MUC16, PDE3A, POM121L2*, and *ZNF165* genes. Genotyping of the same 45 polymorphisms in 264 patients of the Italian CERP study, also treated with opioids for cancer pain, instead confirmed the association for two SNPs in *ZNF568* and *PDE3A* genes. Only one SNP, rs12305038 in *PDE3A*, was confirmed in both series, although with opposite effects of the minor allele on the investigated phenotype. Overall, our findings suggest that genetic factors are indeed associated with nausea and vomiting in opioid-treated cancer patients, but the role of individual polymorphisms may be weak.

## Introduction

Opioid treatment is a standard and consolidated therapy for relieving pain in advanced cancer patients^1^. However, a variable percentage of patients experience poor treatment benefit and also develop side effects^2,3^. Nausea and vomiting occur frequently in opioid therapy for cancer-related pain. A recent systematic review of 25 prospective studies^4^ found that approximately 50% of patients who took morphine, oxycodone, fentanyl, methadone, or hydromorphone for cancer-related pain experienced nausea (ranging from 3% to 85%), whereas the occurrence of vomiting in the same patients ranged from 4% to 50% in the various studies. The wide range in occurrence of nausea and vomiting suggests that genetic factors are involved in individual susceptibility to these side effects.

So far, only a few studies have reported genetic polymorphisms associated with the variability in nausea and vomiting among cancer patients receiving opioids. One multicenter European study that examined polymorphisms in genes related to opioid and nausea/vomiting signaling pathways, in 1579 patients receiving opioids for cancer pain, found associations between these side effects and polymorphisms in *HTR3B*, *COMT*, and *CHRM3* genes^5^. Another study looked specifically at polymorphisms in *CYP2D6*, a gene that encodes an opioid-metabolizing enzyme, and found no effect of *CYP2D6* genotypes on nausea^6^. Both of these studies followed a candidate-gene approach, involving the analysis of known polymorphisms in genes already shown to participate in opioid action or metabolism or in the expression of nausea and vomiting. A candidate-gene approach, which permits the analysis of only a few genes, has major limitations in the study of the genetics of complex phenotypes. Indeed, such phenotypes can be modulated by several molecular players or biochemical pathways, and can be caused by different germline alterations (i.e. they display genetic heterogeneity)^7,8^. Therefore, the selection of genetic candidates based on *a priori* knowledge is limiting and does not always lead to reliable findings^9,10^.

In order to have a more comprehensive view of the genetics of opioid-induced side effects, we carried out a genome-wide association study on individual susceptibility to nausea and vomiting in patients treated with opioids for cancer pain, using the European Pharmacogenetic Opioid Study (EPOS) series^11^ and an independent Italian series from the CERP randomized controlled trial (RCT) of opioids for chronic cancer pain^12^. The objective of this analysis was to identify polymorphisms, throughout the whole exome and not only in candidate genes, that are associated with nausea and vomiting related to the use of opioids in cancer patients.

## Results

To identify genetic variants associated with the intensity of adverse reactions to opioid analgesics, we began by calculating a nausea-vomiting score (NVS) for 1494 cancer patients of the EPOS series (Table 1). These patients were prevalently from Norway, Italy and Germany. The most prevalent cancer types were of the gastrointestinal tract (20%), lung (17%), prostate (11%), breast (9.8%) and female reproductive organs (7.6%). Morphine was the most administered drug, followed by fentanyl and oxycodone. Only 99 EPOS patients (6.6%) received two opioids and one patient received three of them. The patients were grouped according to NVS into those with no or low-intensity reactions (n = 937) and those with moderate or high intensity reactions (n = 557).

**Table 1:** Characteristics of EPOS patients analyzed by exome sequencing and then genotyped to identify genetic variants associated with individual predisposition to nausea and vomiting.

Characteristic	Total	NVS subgroup	
		Pool 1 (<33.3)	Pool 2 (≥33.3)
Patients, n	1494	937	557
Sex, n (%)			
Male	806 (54)	528 (56)	278 (50)
Female	688 (46)	409 (44)	279 (50)
Age, median (range), years	63 (18–91)	63 (18–91)	62 (29–91)
Country, n			
Switzerland	85	50	35
Germany	234	114	120
Denmark	24	21	3
Finland	24	15	9
United Kingdom	195	126	69
Iceland	113	94	19
Italy	297	239	58
Lithuania	41	29	12
Norway	380	191	189
Sweden	101	58	43
Cancer, n (%)			
Gastrointestinal tract^§^	300 (20)	192 (20)	108 (19)
Lung	258 (17)	166 (18)	92 (17)
Prostate	166 (11)	107 (11)	59 (11)
Breast	147 (9.8)	95 (10)	52 (9.3)
Female reproductive organs	114 (7.6)	56 (6.0)	58 (10)
Urological	97 (6.5)	54 (5.8)	43 (7.7)
Head and neck	74 (5.0)	44 (4.7)	30 (5.4)
Other*	338 (23)	223 (24)	115 (21)
Opioid, n (%)			
Morphine	597 (40)	378 (40)	219 (39)
Oxycodone	303 (20)	174 (19)	129 (23)
Fentanyl	433 (29)	290 (31)	143 (26)
Buprenorphine	31 (2)	19 (2)	12 (2)
Other^‡^	130 (9)	76 (8)	54 (10)
Polypharmacy, n (%)			
Only one opioid	1394 (93)	872 (93)	522 (94)
Two opioids	99 (6.6)	64 (6.8)	35 (6.3)
Three opioids	1 (<0.01)	1 (0.11)	0 (0)
NVS, mean (SD)	23.5 (28.2)	5.2 (7.8)	54.4 (22.6)

NVS, nausea-vomiting score; SD, standard deviation.

^§^EPOS did not distinguish colon/rectum from stomach cancers.

*Liver (n = 5), skin (n = 21), pancreas (n = 31), sarcomas (n = 38), multiple cancers (n = 51), unknown (n = 42), not specified (n = 95).

^‡^Hydromorphone (n = 67), ketobemidone (n = 2), levomethadone (n = 25), methadone (n = 35), not reported (n = 1).

Genomic DNA from the EPOS patients was pooled separately for the two groups and used in whole-exome sequencing. A comparison of the two exome sequences identified 53 polymorphisms whose alternative allele fraction differed between the two groups (pool 1 vs. pool 2) by more than 2.5 fold (Supplementary Table S1); such differences were all statistically significant at *P* < 1.0 × 10^−5^. These 53 polymorphisms were all SNPs except for one insertion/deletion (rs66593747). They mapped in 52 unique genes, as both rs1451772 and rs1669412 locate in *TAS2R42* gene. Among these polymorphisms, 29 and 24 showed an enrichment of the variant allele in pool 1 (variant ratio >1) and pool 2 (variant ratio <1), respectively. These polymorphisms were considered potentially associated with the intensity of nausea and vomiting in response to opioids and thus were selected for validation by PCR-based genotyping.

To analyze the 53 candidate polymorphisms by TaqMan OpenArray genotyping, it was necessary to custom-design TaqMan assays for 14 polymorphisms. The design or manufacturing process failed for five SNPs (Supplementary Table S1). Thus, 48 SNP genotyping assays were performed. This analysis was done using individual DNA samples from the same 1494 EPOS patients studied by exome sequencing, and also from 264 patients of the Italian CERP trial (Table 2). This patient series is similar to the EPOS series in terms of sex distribution and administered opioids (only 9% of EPOS patients received an opioid different from the four drugs administered in the CERP trial and no CERP patient received more than one opioid). The two series differ, however, in terms of patients’ nationalities and cancer types, with the most prevalent in CERP being lung cancer, followed by gastrointestinal, breast, gynecologic, head and neck, and pancreas tumors. Moreover, the mean NVS was lower in the CERP series than in the EPOS one (10.2 vs. 23.5): in detail, in the CERP series, only 34 patients (12%) had NVS ≥ 33.3, compared to 557 patients (37%) in the EPOS series.

**Table 2:** Characteristics of 264 CERP patients genotyped to identify genetic factors associated with individual predisposition to nausea and vomiting.

Characteristic	CERP series
Sex, n (%)	
Male	138 (52)
Female	126 (48)
Age, years, median (range)	67.8 (21.2–90.1)
Cancer, n (%)	
Lung	73 (28)
Gastrointestinal tract^§^	42 (16)
Breast	34 (13)
Gynecological tumors	23 (8.7)
Head and neck	21 (8.0)
Pancreas	21 (8.0)
Urological	19 (7.2)
Prostate	12 (4.5)
Other*	19 (7.2)
Concurrent chemotherapy, n (%)	156 (59)
Opioid, n (%)	
Morphine^a^	68 (26)
Oxycodone^a^	61 (23)
Fentanyl^b^	63 (24)
Buprenorphine^b^	72 (27)
NVS, mean (SD)	10.2 (19.4)

NVS, nausea-vomiting score.

^a^Oral; ^b^transdermal administration.

^§^Colon/rectum (n = 34), stomach/esophagus/duodenum (n = 8).

*Myeloma (n = 9), sarcoma (n = 2), unknown (n = 2), not specified (n = 6).

TaqMan genotyping failed for rs35612307 and rs12807084, since genotype clusters were not clearly identifiable. Furthermore, genotyping for rs361498 was non-informative because all individuals of both series were homozygous for the major alleles (not shown); this result, which is discordant with whole-exome sequencing, might be explained by errors in genotype call in one of the two methods used. Thus, 45 SNPs were available for analysis of an association with NVS.

In the EPOS series, we found six SNPs associated with NVS, at a nominal *P* < 0.05 (Table 3). The SNPs showing the best associations were rs168107 and rs12305038, two missense variations in *CLCC1* and *PDE3A* genes, respectively (*P* = 0.001 and *P* = 0.002, respectively). Both these associations showed negative *beta* values, meaning that higher numbers of the minor (variant) alleles of both SNPs (T and A, respectively) associated with lower NVS values. These data are concordant with those from the exome sequencing analysis, where we observed an enrichment of the variant alleles of both SNPs in the pool 1, including individuals with low NVS.

**Table 3:** SNPs associated with NVS phenotype in the EPOS series (n = 1494).

Chromosome	SNP	Position (Mb)*	Gene	Minor allele	Beta^§^	*P* value^§^
1	rs36024412	26.344	AIM1L	T	−2.172	0.042
1	rs168107	108.937	CLCC1	T	−3.545	0.001
6	rs41269255	27.309	POM121L2	T	3.299	0.025
6	rs9393888	28.091	ZNF165	C	−3.071	0.011
12	rs12305038	20.369	PDE3A	A	−3.325	0.002
19	rs11882256	8.950	MUC16	T	2.791	0.016

NVS, nausea-vomiting score.

*Map position, in megabases, based on genome assembly GRCh38.p12.

^§^PLINK, linear regression model of allelic effects on phenotype; covariates: sex, age, country of residence, and opioid type (see Table 1).

In the CERP series, two SNPs associated with NVS: rs12305038 and rs10405238 (*P* = 0.029 and 0.016, respectively; Table 4). One of these markers, rs12305038, associated strongly with NVS in the EPOS series. The minor allele (A) of rs12305038 was the same in both series, but the directions of the associations were opposite, as the *beta* value had a negative and a positive sign in the EPOS and CERP series, respectively. The other marker identified in the CERP series (rs10405238) did not associate with NVS in the EPOS series, and none of the other five SNPs identified in the EPOS series were confirmed in the CERP series.

**Table 4:** SNPs associated with NVS phenotype in 264 CERP patients.

Chromosome	SNP	Position (Mb)	Gene	Minor allele	Beta^§^	*P* value
12	rs12305038	20.369	PDE3A	A	3.982	0.029
19	rs10405238	36.997	ZNF568	G	5.470	0.016

NVS, nausea-vomiting score.

*Map position, in megabases, based on genome assembly GRCh38.p12.

^§^PLINK, linear model of allelic effects on phenotype; covariates: sex, age, and drug.

## Discussion

Using a DNA-pooling approach with whole-exome sequencing, this study preliminarily identified 53 genetic variants throughout the exome whose genotype associated with a composite nausea–vomiting score (NVS) in 1494 cancer patients under opioid analgesia. Individual genotyping of 45 of these candidate polymorphisms, in the same 1494 patients (EPOS series), confirmed the association of six SNPs with NVS. Instead, genotyping of the 45 polymorphisms in 264 patients of the CERP series confirmed the association of two SNPs with NVS. Only one SNP, rs12305038 on chromosome 12, associated with nausea and vomiting phenotype in both series.

The two clinical series studied here, EPOS and CERP, are similar in that both consist of patients with advanced oncological disease causing pain treated with opioid analgesia. The series differ, however, in terms of sample size, prevalent cancer types, and, most importantly, NVS, which was lower in CERP patients. Likely, this last aspect influenced the number of SNPs found to associate with the considered phenotype.

In both series, we found an association of rs12305038 with NVS but with opposite effects of the minor allele, which was associated with less intense side effects in the EPOS series (beta = −3.261) and more intense nausea and vomiting in the CERP series (beta = 3.981). These contradictory statistics could mean that the association is an artifact, but they could also mean that the association is real and represents a functional element in linkage disequilibrium with rs12305038, with different patterns in the two series. Such a possibility is not unprecedented, as alleles having opposite directions of effect in two different populations have been reported in a genetic association study of nicotine dependence^13^.

Our exome-wide study of EPOS cancer patients follows an earlier candidate-gene SNP-genotyping study done in a partially overlapping series of patients. The study reported by Laugsand *et al*.^5^ found significant associations between six polymorphisms in *HTR3B*, *COMT*, and *CHRM3* genes and opioid-related nausea and vomiting, measured as NVS. Those findings were not replicated in the present study, in part because three of the six SNPs identified earlier are intronic polymorphisms that were *a priori* excluded from investigation in this study of exonic variants. For the remaining three SNPs, which do lie in exons, the contrasting results may be attributable to the different study designs. In particular, a limitation of the DNA-pooling approach used here is that it does not permit stratification by covariates, whereas in the candidate-gene study the analyses were stratified by country and use of antiemetics, and several demographic and disease-related variables were used as covariates.

Another difference between the DNA-pooling and individual SNP-genotyping approaches, seen here, lies in the statistical strength of the identified associations. After exome sequencing, the 45 studied SNPs all had a *P* < 1.0 × 10^−5^ in the allelic reconstruction of the results of DNA pools. In contrast, after individual genotyping, the statistical associations of the six confirmed SNPs were much weaker, i.e., with *P* values ranging from 0.040 to 0.001. The poor performance of the technical validation of the exome sequencing results may be due to the DNA pooling design. This strategy saves costs and has been used successfully in other studies^14,15^, which however also reported that it has several limitations (e.g. unequal contributions of different individuals to the DNA pool and variations in sequencing depth in different genes) that can affect the allele frequency estimates. Another limitation is the stochastic loss of rare alleles.

The SNP rs12305038 is a missense variant in the coding portion of the first exon of *PDE3A* gene, which codes for phosphodiesterase 3A. This protein is a member of the class of enzymes that degrade cGMP and cAMP^16^ and therefore are important regulators of cyclic nucleotide signaling in different pathways. Phosphodiesterases (PDEs) are involved in the morphine addiction pathway (hsa05032 KEGG pathway), and also in the bitter and sweet taste signaling pathway^17^. Thus it is possible that the genetic variations in these enzymes that alter taste perception also modulate susceptibility to nausea and vomiting. However, the association of polymorphisms in *PDE3A* gene with nausea and vomiting has never been reported so far.

Genetic variants in *PDE3A* gene have previously been associated with hypertension^18^. Since we did not have access to clinical data regarding the diagnosis of hypertension in the patients in our study, we cannot correct our analysis for this putative confounder. Therefore, we cannot exclude a confounding effect of blood pressure values in the observed association between the SNP in *PDE3A* gene and NVS.

Nausea and vomiting are also side effects of opioid use for postoperative pain, and associations between these side effects and polymorphisms in *FAAH*, *OPRM1*, and *OCT1* genes have been found in several studies that used a candidate-gene investigational approach^19–21^. However, a meta-analysis of studies of opioid therapy for postoperative pain in different clinical settings found that only one polymorphism in the opioid receptor gene *OPRM1* modestly affects opioid-induced nausea and vomiting^22^. In the present exome-wide study, we did not find associations between these polymorphisms and opioid-induced nausea and vomiting in patients with cancer-related pain. These contrasting results may be due to differences in the molecular mechanisms involved in opioid-induced nausea and vomiting in patients with surgical vs. cancer pain. Another possible explanation is the presence of different confounders in the different types of patients. Indeed, some patients in this study were concurrently receiving chemotherapy, which is known to induce nausea and which may have inflated these patients’ NVS values. Also, some gastric cancer patients in our series probably had more severe nausea and vomiting than patients with other cancer types (as suggested by the EORTC QLQ-C30 reference NVS values reported for different cancer types, https://bit.ly/2uDM1pa), thus confounding our results.

Overall, this pharmacogenomics study suggests that the risk of nausea and vomiting in opioid-treated cancer patients has a genetic component. However, the role of individual polymorphisms seems to be weak. Therefore, to clearly identify the genetic elements modulating an individual’s response to opioid treatment, larger and better characterized clinical series are needed, as are individual genotyping or sequencing efforts and new methods for investigating the interactive effects among genetic variants.

## Methods

### Study design and patient selection

**Figure 1:**
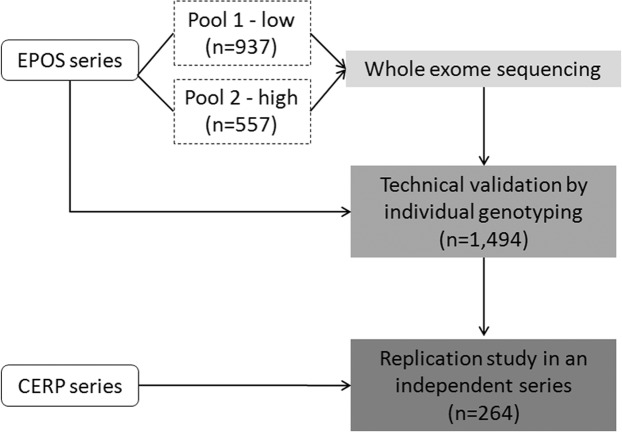
Workflow of the experimental design. The EPOS series was divided in two groups according to the nausea vomiting score (NVS). Pool 1 – low: patients characterized by NVS < 33.3 (no or low nausea and vomiting); Pool 2 – high: patients with NVS ≥ 33.3 (moderate or high nausea and vomiting). DNA pools were genotyped by exome sequencing. Then, the technical validation of 48 selected SNPs was carried out by individual genotyping. Finally, we replicated the study of the selected SNPs in an independent patient series (CERP).

This pharmacogenomic study used a two-part experimental design (Fig. 1), with whole-exome sequencing of two DNA pools (from one clinical series) followed by PCR-based genotyping of individual DNA samples (from two clinical series). The strategy of starting with DNA pools, rather than individual samples, was chosen because of the high costs of individual exome sequencing. This approach has already been used successfully to identify genetic variations associated with other clinical conditions and has been found to be cost effective^14,15^.

For this study, we obtained clinical data and blood or DNA samples from two series of patients that received opioid analgesia for cancer pain. One of these series is EPOS, a cross-sectional, European multicenter study of symptoms, pharmacology, and pharmacogenomics related to the use of opioids (morphine, fentanyl, oxycodone, or other WHO step III opioids^23^) for moderate or severe pain in cancer patients (https://www.ntnu.edu/prc/results/epos). Patient recruitment criteria and measures of nausea and vomiting have been previously described^5,24,25^. The study collected peripheral blood samples from 2305 patients and established a biobank for genetic analysis. For 1494 of these patients, genomic DNA from peripheral blood was available for use in this study (as described in^25^).

The other clinical series investigated here is from the CERP study^12^, a four-arm, Italian multicenter RCT that included 520 patients with moderate to severe cancer pain requiring WHO step III opioids. In this trial, patients had been randomly assigned to receive oral morphine, oral oxycodone, transdermal buprenorphine, or transdermal fentanyl for 28 days. For 264 of these patients, peripheral blood samples were available for use in this study.

The study protocol was approved by the Committees for Ethics of each recruiting hospital contributing to the EPOS and CERP studies (a full list of all the ethics committees is available in the Supplementary Information), and the research was conducted in accordance with the tenets of the Declaration of Helsinki. All patients provided informed consent for the collection of clinical information and biological materials for research purposes.

### Nausea and vomiting phenotype

The phenotype under study, i.e. the presence of nausea and vomiting, was assessed using two different patient-reported outcome measures (PROMs). The European Organization for Research and Treatment of Cancer’s Core Quality of Life Questionnaire (EORTC QLQ-C30)^26,27^ was used by EPOS, while the Therapy Impact Questionnaire (TIQ)^28^ was used by CERP. In both PROMs, nausea and vomiting intensities were assessed on four-point verbal rating scales. For QLQ-C30, the four points are “not at all”, “a little”, “quite a bit”, and “very much”, and they are assigned numerical values from 1 to 4. Similarly, for TIQ, the four points are “not at all”, “some”, “a lot”, and “very much”. EPOS patients did the questionnaire at least 24 h from taking chemotherapy, while CERP patients did it concurrently with non-first-line chemotherapy (59% of the CERP series) but at least 14 days after antalgic radiotherapy.

Following the procedure described in the EORTC QLQ-C30 Scoring Manual^29^ we calculated the mean of the QLQ-C30 scores for nausea and vomiting, and then linearly transformed this raw score into a composite nausea–vomiting score (NVS) that ranges from 0 to 100, with higher values indicating higher impairment. Given the similarity between QLQ-C30 and TIQ, we used the same procedure for TIQ and considered the two NVSs to be equivalent. On the basis of our clinical experience, we chose an NVS cutoff of 33.3 to distinguish patients who had no or low-intensity nausea or vomiting (NVS < 33.3) from those with moderate or high intensity (NVS ≥ 33.3). We did not use the EORTC QLQ-C30 reference value for NVS in recurrent/metastatic cancer patients (NVS = 13.1, https://bit.ly/2uDM1pa) to dichotomize our study group, since it was much lower than the EPOS series’ mean NVS.

### DNA pools and whole exome-sequencing

We divided the 1494 EPOS patients into two groups characterized by NVS < 33.3 (no or low nausea and vomiting) *versus* NVS ≥ 33.3 (moderate or high nausea and vomiting). Equal amounts of genomic DNA from each patient in the two groups were combined to create two DNA pools.

Each DNA pool (50 ng) was used to prepare an exome library according to the standard Nextera Rapid Capture Exome Kit (Illumina) protocol. DNA fragments ranging from 250 bp to 350 bp were selected on 2% agarose gels. The concentration of each library was determined using a 2100 Bioanalyzer (Agilent), which also verified the sequence-grade quality of the DNA. The libraries were then sequenced on a HiSeq2500 system (Illumina) in “rapid run” mode with 100 bp-long paired-end reads.

Paired FASTQ sequence data were aligned to the human reference genome (GRCh37/hg19) using Burrows-Wheeler alignment (BWA) tool^30^. Specifically, the BWA *aln* algorithm (http://bio-bwa.sourceforge.net/bwa.shtml) was used to align the sequences with the –n parameter set to 0.04, where n represents the fraction of missing alignments given a 2% uniform base error rate. Paired reads were then processed using *sample* with the following parameters: -a 500 -o 100 -N 10. SAM files were converted to BAM format, sorted and indexed using SAMtools^31^ with default settings. Polymorphisms differentially present in the two groups were identified using CEQer2, an updated version of CEQer^32^, as described^33^. Filtering criteria were: p ≤ 0.05; alternative allele fraction (i.e. number of reads carrying an alternative allele at a certain position, divided by the total number of reads for that position) for each pool, ≥0.35; and alternative allele fraction ratio in pool 1/pool 2 ≤ 0.4 or ≥2.5.

### SNP validation by individual genotyping

Polymorphisms identified by exome sequencing of DNA pools were genotyped in individual samples of the same EPOS series to technically replicate the findings. Additionally, we genotyped these same polymorphisms in the CERP patients to confirm the results in an independent series. For this purpose, genomic DNA was extracted from peripheral blood of each CERP patient, using the DNeasy Blood & Tissue kit (Qiagen) and fluorimetrically quantified. Then, already available and custom-ordered TaqMan SNP Genotyping Assays were used on the TaqMan OpenArray Genotyping System (Thermo Fisher Scientific). DNA samples (from both EPOS and CERP series) were loaded at 50 ng/mL and amplified according to the standard protocol. Genotypes were determined used auto-calling methods, as implemented in the Genotyping analysis module, version 3.3, of the Applied Biosystems analysis software, on the Thermo Fisher Cloud. A genotype call rate ≥0.90 was selected as the reliability threshold; this means that SNPs for which it was impossible to determine the genotype in more than 10% of samples were not considered in the subsequent analysis.

### Statistical analysis

In the technical validation of exome sequencing results (EPOS series), to identify genetic variants associated with nausea and vomiting, we used a linear regression model with NVS as the dependent variable and sex, age, country of residence, and type of opioid as covariates. In the replication series (CERP), we used a linear regression model with NVS as the dependent variable and sex, age, and drug as covariates, to identify genetic variants associated with nausea and vomiting. In both analyses, a positive *beta* value means that the higher is the number of copies of the minor allele of any polymorphism, the higher is the quantitative value of the phenotype on study, i.e., nausea and vomiting intensity. These analyses were done using PLINK software^34^. A value of *P* < 0.05 indicated statistical significance.

## Supplementary information

Supplementary Table S1 and Information.

## References

[1] Caraceni A (2012). Use of opioid analgesics in the treatment of cancer pain: evidence-based recommendations from the EAPC. Lancet Oncol..

[2] Teunissen SC (2007). Symptom prevalence in patients with incurable cancer: a systematic review. J. Pain Symptom Manage..

[3] Klepstad P (2005). Pain and pain treatments in European palliative care units. A cross sectional survey from the European Association for Palliative Care Research. Network. Palliat. Med..

[4] Oosten AW, Oldenmenger WH, Mathijssen RH, van der Rijt CC (2015). A Systematic Review of Prospective Studies Reporting Adverse Events of Commonly Used Opioids for Cancer-Related Pain: A Call for the Use of Standardized Outcome Measures. J. Pain.

[5] Laugsand EA (2011). Clinical and genetic factors associated with nausea and vomiting in cancer patients receiving opioids. Eur. J. Cancer.

[6] Andreassen TN (2012). Do CYP2D6 genotypes reflect oxycodone requirements for cancer patients treated for cancer pain? A cross-sectional multicentre study. Eur. J. Clin. Pharmacol..

[7] Galvan A, Ioannidis JP, Dragani TA (2010). Beyond genome-wide association studies: genetic heterogeneity and individual predisposition to cancer. Trends Genet..

[8] Roberts NJ (2016). Whole Genome Sequencing Defines the Genetic Heterogeneity of Familial Pancreatic Cancer. Cancer. Discov..

[9] Low SK, Takahashi A, Mushiroda T, Kubo M (2014). Genome-wide association study: a useful tool to identify common genetic variants associated with drug toxicity and efficacy in cancer pharmacogenomics. Clin. Cancer Res..

[10] Tizaoui, K. Multiple sclerosis genetics: Results from meta-analyses of candidate-gene association studies. *Cytokine* (2017). 10.1016/j.cyto.2017.10.024 29103823

[11] Kurita GP (2011). Prevalence and predictors of cognitive dysfunction in opioid-treated patients with cancer: a multinational study. J. Clin. Oncol..

[12] Corli O (2016). Are strong opioids equally effective and safe in the treatment of chronic cancer pain? A multicenter randomized phase IV ‘real life’ trial on the variability of response to opioids. Ann. Oncol..

[13] Saccone NL (2010). Multiple cholinergic nicotinic receptor genes affect nicotine dependence risk in African and European Americans. Genes Brain Behav..

[14] Wang J (2016). Investigation of rare and low-frequency variants using high-throughput sequencing with pooled DNA samples. Sci. Rep..

[15] Kaartokallio T (2016). Exome sequencing in pooled DNA samples to identify maternal pre-eclampsia risk variants. Sci. Rep..

[16] Francis SH, Blount MA, Corbin JD (2011). Mammalian cyclic nucleotide phosphodiesterases: molecular mechanisms and physiological functions. Physiol. Rev..

[17] Amrein H, Bray S (2003). Bitter-sweet solution in taste transduction. Cell.

[18] Kato N (2015). Trans-ancestry genome-wide association study identifies 12 genetic loci influencing blood pressure and implicates a role for DNA methylation. Nat. Genet..

[19] Sadhasivam S (2015). Novel associations between FAAH genetic variants and postoperative central opioid-related adverse effects. Pharmacogenomics J..

[20] Sugino S (2014). Association of mu-opioid receptor gene (OPRM1) haplotypes with postoperative nausea and vomiting. Exp. Brain Res..

[21] Balyan R (2017). OCT1 genetic variants are associated with postoperative morphine-related adverse effects in children. Pharmacogenomics.

[22] Ren ZY (2015). The impact of genetic variation on sensitivity to opioid analgesics in patients with postoperative pain: a systematic review and meta-analysis. Pain Physician..

[23] World Health Organization. WHO. Cancer pain relief. 2nd ed. *World Health Organization, Geneva* (1996).

[24] Knudsen, A. K. *et al*. Which variables are associated with pain intensity and treatment response in advanced cancer patients? - Implications for a future classification system for cancer pain. *Eur. J. Pain* (2010). 10.1016/j.ejpain.2010.08.001 20822941

[25] Galvan A (2011). Multiple Loci modulate opioid therapy response for cancer pain. Clin. Cancer Res..

[26] Aaronson NK (1993). The European Organization for Research and Treatment of Cancer QLQ-C30: a quality-of-life instrument for use in international clinical trials in oncology. J. Natl. Cancer Inst..

[27] Bjordal K (2000). A 12 country field study of the EORTC QLQ-C30 (version 3.0) and the head and neck cancer specific module (EORTC QLQ-H&N35) in head and neck patients. EORTC Quality of Life Group. Eur. J. Cancer.

[28] Tamburini M (1992). A therapy impact questionnaire for quality-of-life assessment in advanced cancer research. Ann. Oncol..

[29] Fayers, P. M. *et al*. The EORTC QLQ-C30 Scoring Manual (3rd Edition). *European Organisation for Research and Treatment of Cancer, Brussels* (2001).

[30] Li H, Durbin R (2009). Fast and accurate short read alignment with Burrows-Wheeler transform. Bioinformatics.

[31] Li H (2009). The Sequence Alignment/Map format and SAMtools. Bioinformatics.

[32] Piazza R (2013). CEQer: a graphical tool for copy number and allelic imbalance detection from whole-exome sequencing data. PLoS One.

[33] Piazza R (2013). Recurrent SETBP1 mutations in atypical chronic myeloid leukemia. Nat. Genet..

[34] Purcell S (2007). PLINK: a tool set for whole-genome association and population-based linkage analyses. Am. J. Hum. Genet..

