# Supplementary material for: Identification of genetic polymorphisms modulating nausea and vomiting in two series of opioid-treated cancer patients
Source: Sci Rep. 2020 Jan 17;10:542. doi: 10.1038/s41598-019-57358-y (PMC6969029; doi:10.1038/s41598-019-57358-y)
Supplement: Supplementary file 1 — Supplementary Table S1 and Information. [file 41598_2019_57358_MOESM1_ESM.docx]

Identification of genetic polymorphisms modulating nausea and vomiting in two series of opioid-treated cancer patients

Francesca Colombo ^1^, Giulia Pintarelli ^1^, Antonella Galvan ^1^, Sara Noci ^1^, Oscar Corli ^2^, Frank Skorpen ^3,4^, Pål Klepstad ^3,5^, Stein Kaasa ^3,6^, Alessandra Pigni ^1^, Cinzia Brunelli ^1,3,4^, Anna Roberto ^2^, Rocco Piazza ^7^, Alessandra Pirola ^7^, Carlo Gambacorti-Passerini ^7^, Augusto Caraceni ^1,3^

^1^ Fondazione IRCCS Istituto Nazionale Tumori, Milan, Italy;

^2^ Pain and Palliative Care Research Unit, IRCCS Istituto di Ricerche Farmacologiche Mario Negri, Milan, Italy;

^3^ European Palliative Care Research Center, and

^4^ Department of Clinical and Molecular Medicine, Faculty of Medicine and Health Sciences, Norwegian University of Science and Technology, Trondheim, Norway

^5^ Department of Anesthesiology and Emergency Medicine and

^6^ Department of Oncology, St. Olavs University Hospital, Trondheim, Norway

^7^ Department of Medicine and Surgery, University of Milano-Bicocca, Monza, Italy

**Supplementary Table S1**. Characteristics of 53 polymorphisms identified by whole-exome sequencing as having different alternative allele fractions between EPOS patients having low (NVS<33.3) and high (NVS≥33.3) intensity of nausea and vomiting in response to opioid analgesia for cancer pain. Data are sorted according to variant ratio (pool 1 / pool 2)

| **Polymorphism** | **Chr.** | **Position** | **Gene** | **Reference allele** | **Variant allele** | **Alternative allele fraction ratio**  **(pool 1 / pool 2)** | **TaqMan assay ^^^ for individual sample genotyping** |
| --- | --- | --- | --- | --- | --- | --- | --- |
| rs2241575 | 11 | 129,784,456 | PRDM10 | G | A | 18.85 | C__26489565_10 |
| rs3743588 | 16 | 11,836,508 | TXNDC11 | G | A | 11.61 | manufacturing failed |
| rs877834 | 7 | 25,267,934 | NPVF | T | C | 9.47 | C___2559446_1_ |
| rs12305038 | 12 | 20,522,252 | PDE3A | G | A | 5.99 | C__25600078_20 |
| rs35612307 ^§^ | 19 | 14,875,388 | EMR2 | G | A | 4.85 | C__33040957_10 |
| rs1135840 | 22 | 42,522,613 | CYP2D6 | G | C | 4.42 | C__27102414_10 |
| rs2272996 | 6 | 133,015,271 | VNN1 | T | C | 4.29 | C__25472082_10 |
| rs7224496 | 17 | 1,704,296 | SMYD4 | C | A | 4.24 | C__11626716_10 |
| rs11880955 | 19 | 14,952,017 | OR7A10 | T | A | 4.11 | C__30918832_10 |
| rs12807084 * | 11 | 1,018,144 | MUC6 | G | C | 3.80 | C__45144909_10 |
| rs33990382 | 13 | 24,876,752 | SPATA13 | T | C | 3.76 | C__11499953_10 |
| rs62490888 | 8 | 11,418,766 | BLK | G | A | 3.39 | AH6R653 |
| rs11882256 | 19 | 9,060,541 | MUC16 | C | T | 3.35 | C__25745460_10 |
| rs3811987 | 5 | 74,324,548 | GCNT4 | G | A | 3.28 | C__26555228_10 |
| rs36024412 | 1 | 26,670,444 | AIM1L | G | T | 3.10 | C____507607_10 |
| rs527176 | 2 | 23,865,908 | KLHL29 | G | C | 2.95 | C___2397315_10 |
| rs168107 | 1 | 109,479,978 | CLCC1 | G | T | 2.89 | C____582924_1_ |
| rs2152143 | 10 | 129,906,980 | MKI67 | C | T | 2.80 | C___1801147_10 |
| rs1453541 | 11 | 59,225,221 | OR4D6 | T | C | 2.77 | C___8141118_10 |
| rs10771894 | 12 | 32,134,943 | KIAA1551 | A | G | 2.76 | C__25641490_20 |
| rs10405238 | 19 | 37,488,055 | ZNF568 | T | G | 2.76 | C__25998429_10 |
| rs361498 * | 17 | 25,970,642 | LGALS9 | A | T | 2.72 | AHD2C60 |
| rs361360 | 7 | 142,247,213 | TCRBV6S1A1N1 | G | T | 2.72 | C__26657030_10 |
| rs9438 | 3 | 154,018,887 | DHX36 | G | C | 2.72 | C___1037494_10 |
| rs235836 | X | 48,418,659 | TBC1D25 | G | A | 2.65 | AHFBBC8 |
| rs3739038 | 2 | 238,672,703 | LRRFIP1 | C | G | 2.64 | AHD2DA8 |
| rs3766163 | 1 | 15,986,547 | DDI2 | T | C | 2.61 | C__25760835_10 |
| rs12226920 | 12 | 11,150,046 | TAS2R20 | G | T | 2.51 | C___1326611_10 |
| rs55641679 | 11 | 1,093,582 | MUC2 | G | C | 2.51 | manufacturing failed |
| rs4804401 | 19 | 9,213,079 | OR7G2 | A | C | 0.40 | C__11695723_10 |
| rs72885464 | 2 | 171,570,151 | LOC440925 | C | T | 0.39 | AHFBBHG |
| rs3763384 | 7 | 4,876,057 | RADIL | C | T | 0.39 | C___1181168_10 |
| rs66593747 | 7 | 137,612,914 | CREB3L2 | T | -TGG | 0.38 | AHHS7PO |
| rs1190788 | 6 | 131,520,655 | AKAP7 | G | A | 0.38 | C___1649707_10 |
| rs1057463 | 8 | 67,592,152 | C8orf44 | T | C | 0.36 | C___1423285_10 |
| rs76615008 | 11 | 7,817,852 | OR5P2 | C | G | 0.36 | manufacturing failed |
| rs156697 | 10 | 106,039,185 | GSTO2 | A | G | 0.35 | C___3223136_1_ |
| rs62282474 | 3 | 195,508,091 | MUC4 | T | C | 0.34 | design failed |
| rs11736872 | 4 | 177,100,644 | WDR17 | G | A | 0.33 | C____379778_10 |
| rs4589164 | 1 | 169,256,501 | NME7 | C | T | 0.32 | C__11341772_10 |
| rs28502153 | 22 | 17,469,049 | GAB4 | C | A | 0.32 | C__25932150_10 |
| rs12610094 | 19 | 9,213,132 | OR7G2 | A | G | 0.31 | C__11695724_10 |
| rs801840 | 7 | 88,964,196 | ZNF804B | T | A | 0.31 | manufacturing failed |
| rs9648725 | 7 | 151,504,019 | BC040865 | T | C | 0.3 | AH705CB |
| rs1669412 | 12 | 11,338,669 | TAS2R42 | C | T | 0.3 | AH893IJ |
| rs1451772 | 12 | 11,338,750 | TAS2R42 | T | C | 0.29 | AH5I8ZV |
| rs35385129 | 19 | 45,162,189 | PVR | C | A | 0.26 | C___1828143_10 |
| rs9393888 | 6 | 28,059,217 | LOC100129195 | G | C | 0.22 | C____233059_10 |
| rs2290228 | 7 | 128,388,648 | CALU | G | A | 0.21 | C___1327891_1_ |
| rs41269255 | 6 | 27,277,051 | POM121L2 | C | T | 0.18 | C__25641021_10 |
| rs11083857 | 19 | 47,549,454 | TMEM160 | C | T | 0.17 | C__31006560_10 |
| rs520805 | 11 | 121,000,774 | TECTA | T | C | 0.17 | C___1316395_10 |
| rs4816 | 6 | 150,114,745 | PCMT1 | G | A | 0.10 | C__11415888_10 |

NVS, nausea-vomiting score; Chr., chromosome

^ TaqMan SNP Genotyping Assays (commercially available or custom) for TaqMan OpenArray Genotyping

§ OpenArray genotyping failed

* Non-informative SNPs in OpenArray genotyping

**List of the Committees for Ethics of each recruiting hospital contributing to the EPOS and CERP studies**

*EPOS study*

The Scientific Ethics Committees for the municipalities of Copenhagen and Frederiksberg, Denmark

Ethics committee of the Medicine Faculty of the University Hospital RWTH Aachen, Germany

[The National Bioethics Committee](https://www.vsn.is/en), Iceland

Independent Ethics Committee of the Istituto Nazionale Tumori, Milan, Italy

Ethics Committee of the Azienda-USL Forlì, Morgagni-Pierantoni Hospital, Forlì, Italy

Ethics Committee of the Fondazione Salvatore Maugeri, Pavia, Italy

Lithuanian Bioethics Committee, Lithuania

NTNU Norwegian University of Science and Technology, Faculty of Medicine, Regional Medical Research Ethics Committee, Central Norway Health Authority, Norway

Regional Ethics Review Board, Stockholm, Sweden

Ethics Committee of the Canton St. Gallen, Switzerland

The Royal Marsden Local Research Ethics Committee, United Kingdom

*CERP study*

Ethics Committee for Clinical Drug Experimentation of AUSL of Pescara, Italy

Ethics Committee of the Università Degli Studi Gabriele D`Annunzio and of the ASL 2 Lanciano-Vasto-Chieti, Italy

Ethics Committee of the A.O. Bianchi-Melacrino-Morelli, Reggio Calabria, Italy

Ethics Committee of the IRCCS Istituto Nazionale per lo studio e la cura dei Tumori, Fondazione Giovanni Pascale, Naples, Italy

Ethics Committee of the A.O. V. Monaldi, Naples, Italy

Ethics Committee of the A.O. Antonio Cardarelli, Naples, Italy

Bioethics Committe of the San Pietro hospital, Rome, Italy

Independent Ethics Committee of the A.O.U. Policlinico S. Orsola-Malpighi, Bologna, Italy

Ethics Committee of the Province of Modena, Italy

Unique Ethics Committee of the Province of Parma, Italy

Ethics Committee of the Province of Reggio Emilia, Italy

Ethics Committee of the AUSL of Piacenza, Italy

Ethics Committee of the Area Vasta Romagna and Istituto Scientifico Romagnolo per lo studio e la cura dei Tumori of Meldola, Italy

Ethics Committee of the Azienda Policlinico Umberto I, Rome, Italy

Independent Ethics Committee of the Fondazione Policlinico Tor Vergata, Rome, Italy

Ethics Committee of the AUSL of Viterbo, Italy

Ethics Committee of the ASL of Frosinone, Italy

Ethics Committee of the ASL 3 Genovese, Genoa, Italy

Ethics Committee of the Ente Ospedaliero Ospedali Galliera, Genoa, Italy

Ethics Committee of the A.O. Universitaria S. Martino, Genoa, Italy

Ethics Committee of the Fondazione Salvatore Maugeri, Pavia, Italy

Ethics Committee of the A.O. Ospedale di Circolo of Busto Arsizio, Italy

Ethics Committee of the A.O. G. Salvini of Sesto San Giovanni, Italy

Ethics Committee of the Istituti Clinici Di Perfezionamento, Milan, Italy

Ethics Committee of the A.O.S. Paolo, Milan, Italy

Ethics Committee of the IRCCS Multimedica, Sesto San Giovanni, Italy

Ethics Committee of the Ospedale Maggiore Policlinico Milano, Italy

Ethics Committee of the Fondazione S. Raffaele del Monte Tabor, Milan, Italy

Independent Ethics Committee of the Istituto Nazionale Tumori, Milan, Italy

Independent Ethics Committee of the A.O. Carlo Poma, Mantova, Italy

Ethics Committee of the A.O. della Valtellina e della Valchiavenna, Sondrio, Italy

Ethics Committee of the ASUR Zona Territoriale 8 of Civitanova Marche and Zona Territoriale 9, Macerata, Italy

Ethics Committee of the A.O.U. S. Giovanni Battista, Torino, Italy

Ethics Committee of the ASL To/2 of Torino, Italy

Scientific Ethics Committee of the A.O.U. Policlinico G. Martino, Messina, Italy

Ethics Committee of the A.O.U. Policlinico-Vittorio Emanuele, Catania, Italy

Ethics Committee of the Azienda Sanitaria Provinciale of Trapani, Italy

Ethics Committee for Clinical Drug Experimentation of Province of Venice, Italy
